# Supplementary material for: Endo-β-1,3-glucanase (GH16 Family) from Trichoderma harzianum Participates in Cell Wall Biogenesis but Is Not Essential for Antagonism Against Plant Pathogens
Source: Biomolecules. 2019 Nov 26;9(12):781. doi: 10.3390/biom9120781 (PMC6995588; doi:10.3390/biom9120781)
Supplement: Supplementary file 1 [file biomolecules-09-00781-s001.pdf]

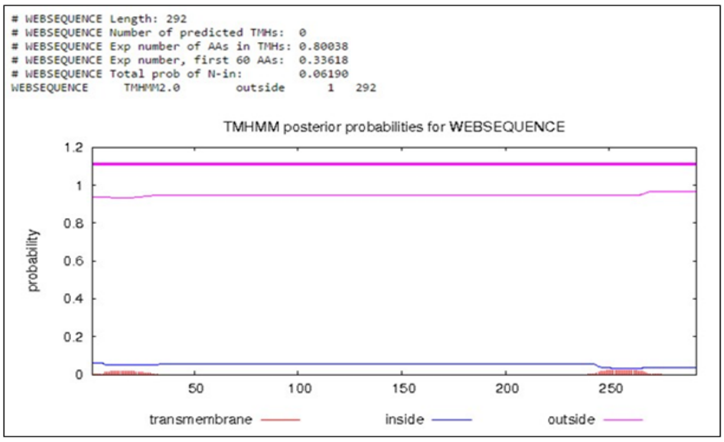

Supplementary Figure 1. Prediction of a probable transmembrane helices localization and orientation. The vertical line (red) indicates the probable transmembrane sites; line parallel to x-axis indicates the probable sites membrane inside (blue) or membrane outside (pink).

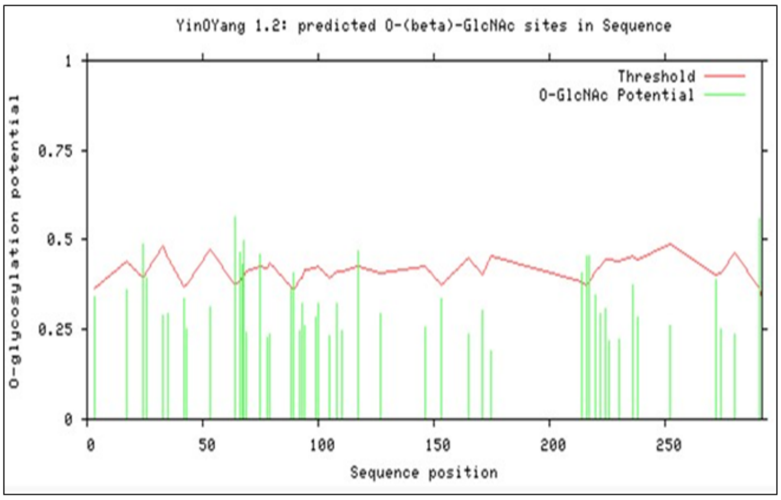

Supplementary Figure 2. O-β-GlcNAc prediction of the β-1,3-glucanase aminoacid sequence. Thex-axis represents to N-terminal sequence for C-terminal.Vertical lines(green) are potential O-GlcNAc sites. The threshold potential is representes by the horizontal line (red). The site with vertical lines (green) that crossed the threshold line are consideres site predictes for O- GlcNAc sites.

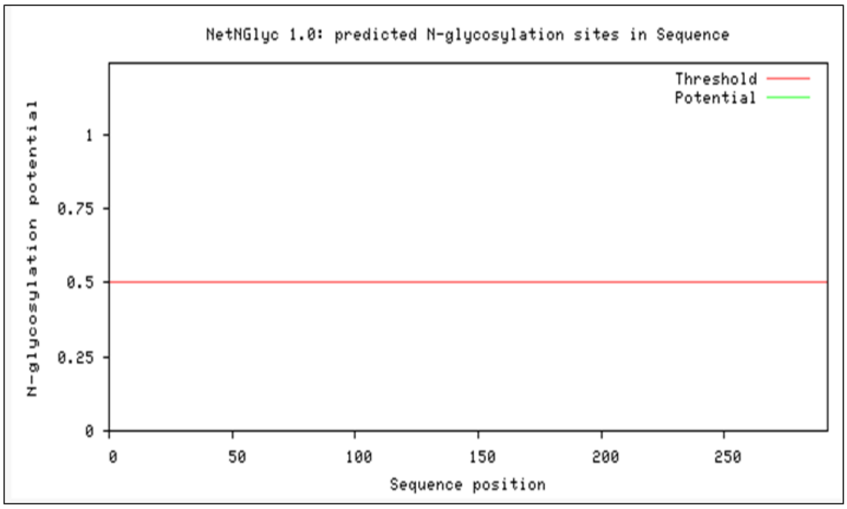

Supplementary Figure 3. Prediction *N*-glic sites of the β-1,3-glucanase aminoacid sequence. X-axis represents protein length from N- to C-terminal. Aposition with apotential (verticalline) crossing the threshold line (red) is predicted glycosylated.

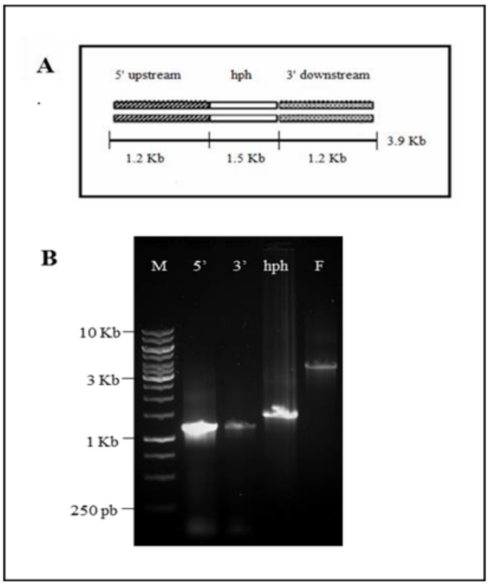

Supplementary Figure 4. Deletion cassette of *T.harzianum* glucanase.**(A).**Scheme of deletion cassette showing expected size for each region separately and after fragments fusion. **(B)**. Evaluation of size amplified fragments in 1% agarose gel. M: 1Kb molecular marker; 5': flanking region 5' usptream; 3 ': flanking region 3' downstream; F: fusion product of three fragments (deletion cassette).

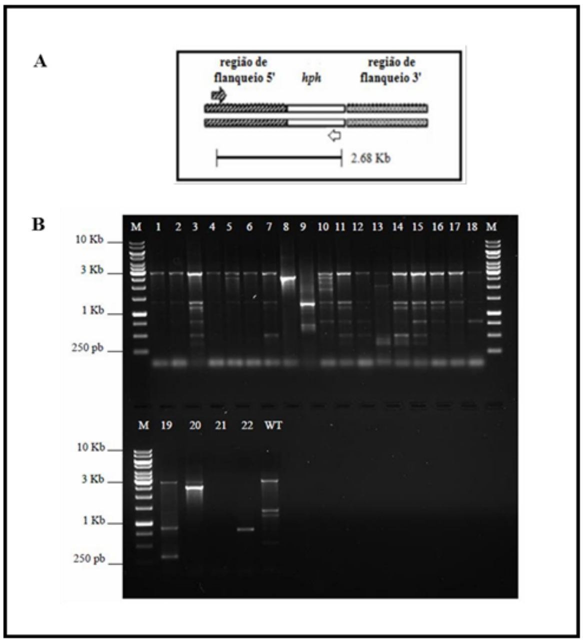

Supplementary Figure 5. (A). Analysis of transformants by PCR. Strategy used to confirm PCR replacement of the *gluc31* gene by the hph marker gene. For amplification of the 2.68 Kb fragment,specific primers were used for the 5'upstream region and for the hygromycin region. **B).** Fragments amplified in 1% agarose gel. Transformant 8 and 20 indicated the expected amplification size. M: 1Kb molecular marker; 1 to 22: transformants; WT: wildlineage.

Supplementary Tabela S1: Primers used in the deletion strategy of the *gluc31* gene of *T. harzianum.*

Gene	Oligonucleo-tídeos	Sequencia	Amplicon (pb)
Endoglucanase	Del_5F	CTCCGGGGCGGCAATTCCCAAGTTGATAGTAC	1214
	Del_5R	ATCCACTTAACGTTACTGAAATCTCCAACGCCATTGTCATCCGTGATA	
	Del_3F	CTCCTTCAATATCATCTTCTGTCTCCGACCATGCGATACCACAGTACATAA	1214
	Del_3R	GGATACCTGGTTGACCCGGAGAGGCG	
	Nested 5R	GTGATGGAAAGATGGTGTGGAG	3494
	Nested 3F	CGCAGGGTAACTAGCTACAACAT	
Higromicina	Hyg_5F	GTCGGAGACAGAAGATGATATTGAAGGAGC	1432
	Hyg_3R	GTTGGAGATTTCAGTAACGTTAAGTGGAT	

Supplementary Tabela S2. Primers used in Gene Expression Analysis by Real-Time PCR.

Gene	Primer	Sequence
Endoglucanase GH16	150678 F	GCCAACAAGCAGGGTATCT
	150678 R	GATGTCGATCTCACCACAGTT
	101233 R	GTC AAT CTC CTG GGT GTC ATT
	101233 F	GCG AGG AAG GAC TTG CAT TA
	513507 R	CCG CTG CTA CGA TAG TTG TT
	513507 F	CAC TGG CGG AGC TAT GAT ATT
	99201 R	GGC AGG TTG TTC CAC TTC T
	99201 F	GGG AGA GCA TCA AGG TTA CTG
	513340 R	TTT GGA TAC CAG GCG TTG AG
	513340 F	GGC CGC TAT CTC ATT CTA CAT C
	512534 R	GAG TCC AAG ACT GGA TGG ATT C
	512534 F	AAC CAG GAG CTC GAG ACT TA
	486218 R	CAC CTC CTT GGT CCA AAT ACT C
	486218 F	CTG GCT ATT TCC GGC TTC TT
	84167 R	CAG ACT TGA GGA CAA TAG CAG TAA
	84167 F	CCC ACT CTA ACC AGC AAC AA
	15669 R	TTG GAA CCT CAC CCT TCA TTA C
	15669 F	GCC GTC CTG TAC GAT ATG ATT T
	15000 R	GAA GAG TCG CTG CTG TCA T
	15000 F	CAA GAG CGT CAA GGA GTA CAT
	78123 R	CTC GTA ATG TCA GAG GGA ATG G
	78123 F	TGG CTC GGA TTA CAA CAA GAA
	2738 R	CGT ACA CAT CGA TCT CTC CAT C
	2738 F	CCC ACC TGC CTA CCA ATA AG
	503986 R	GCA CCT AGC CAC TCC AAA T
	503986 F	CGT GTT GAA GTC ACC ATG AAA G
	1670 R	TCC CGG CAT ATG AGC AAT ATC
	1670 F	TTA TGG GCG TCG ATT ACA CTA C
	503766 R	GTG GCA TCT CGG TAT GTG AA
	503766 F	CAG ACA AAG ACG TCG GAC TAC
	503062 R	GCC ACC TCA GTA ATC GTC TTA G
	503062 F	TAC TGT GTA CGG CTG TGT TG
	97567 R	CGG ATG TTG TCG TAG GTG TAG
	97567 F	CAT CCA GAA GAA CGG CTA CTT
Chitynsinthase	766590_ThF	GGCTATATGGCGTTATCCTCATC
	766590_ThR	CCAGACCATGGCCGAATAAA
Actin	Actin_F	GACATTTACGGCGGAGATGAG
	Actin_R	AGGACGGCAGGATTGATTTG

Supplementary Table S3. Evaluation of antagonistic potential of T.harzianum wild and mutante strains against diferente plant pathogens, according to Bell et al.,1982.

Lineages	*R. solani*	*S. sclerotiorum*	*F. oxysporum*
T. harzianum(wt)	1 a	3 a	3 a
T. harzianum(*Δgluc31*)	1 a	3 a	3 a
